# Rapid Genomic Characterization of SARS-CoV-2 by Direct Amplicon-Based Sequencing Through Comparison of MinION and Illumina iSeq100^TM^ System

**DOI:** 10.3389/fmicb.2020.571328

**Published:** 2020-09-25

**Authors:** Véronique Hourdel, Aurelia Kwasiborski, Charlotte Balière, Séverine Matheus, Christophe Frédéric Batéjat, Jean-Claude Manuguerra, Jessica Vanhomwegen, Valérie Caro

**Affiliations:** Environment and Infectious Risks Unit, Institut Pasteur, Paris, France

**Keywords:** COVID-19, real-time genome sequencing, amplicon-based sequencing, iSeq100^TM^ system, MinION, SARS-CoV-2

## Abstract

Global human health is increasingly challenged by emerging viral threats, especially those observed over the last 20 years with coronavirus-related human diseases, such as the Severe Acute Respiratory Syndrome (SARS) and the Middle East Respiratory Syndrome (MERS). Recently, in late December 2019, a novel *Betacoronavirus*, SARS-CoV-2, originating from the Chinese city of Wuhan, emerged and was then identified as the causative agent of a new severe form of pneumonia, COVID-19. Real-time genome sequencing in such viral outbreaks is a key issue to confirm identification and characterization of the involved pathogen and to help establish public health measures. Here, we implemented an amplicon-based sequencing approach combined with easily deployable next-generation sequencers, the small and hand-held MinION sequencer and the latest most compact Illumina sequencer, the iSeq100^TM^ system. Our results highlighted the great potential of the amplicon-based approach to obtain consensus genomes of SARS-CoV-2 from clinical samples in just a few hours. Both these mobile next-generation sequencers are proven to be efficient to obtain viral sequences and easy to implement, with a minimal laboratory environment requirement, providing useful opportunities in the field and in remote areas.

## Introduction

Global human health is increasingly challenged by emerging viral threats, especially those observed over the last 20 years with the Severe Acute Respiratory Syndrome (SARS) and the Middle East Respiratory Syndrome (MERS) caused by coronaviruses. Recently, in late December 2019, a novel *Betacoronavirus*, SARS-CoV-2, originating from Wuhan, Hubei province, China, emerged and was identified as the causative agent of a new form of severe pneumonia, named coronavirus disease 2019 (COVID-19) (Wu et al., 2020; Zhou et al., 2020). The outbreak spread further to Europe and America mid-March 2020 to become a global public health emergency, with the vast majority of countries and territories reporting confirmed cases. By June 2, 2020, the number of confirmed cases in the world has increased to over 6,306,746 (Dong et al., 2020). SARS-CoV-2, an enveloped positive-sense (+) single-stranded (ss) RNA virus, is a new member of the *Coronaviridae* family, *Betacoronavirus* genus, and the seventh coronavirus known to infect humans. Genetically distinct from SARS-CoV and MERS-CoV, SARS-CoV-2 was included in the monophyletic clade 2, within subgenus *Sarbecovirus*, due to its closest sequence similarity with Chinese Bat SARS-like coronaviruses (Lu et al., 2020; Wu et al., 2020; Ye et al., 2020). Despite its RNA proofreading capacity during replication like all members of the *Coronaviridae* family, SARS-CoV-2 could nevertheless acquire some nucleotide mutations along the genome, allowing for tracking its spread and thus clade definition.

The implementation of short turnaround time and user-friendly sequencing tools in affected regions to track the spread, analyze variants, and identify clusters of transmission of emerging viruses, like SARS-CoV-2, is becoming essential. Although whole genome sequencing of viral pathogens has become a routine procedure in epidemiological monitoring and surveillance, these molecular investigations essential for public health support are still challenging in remote areas with poor technical resources. Thus, the development of easily deployable viral genome toolkits can be proposed to rapidly obtain SARS-CoV-2 genomic sequence data in the field without the requirement to send biological samples to suitably equipped and reference laboratories. These molecular investigations also depend on efficient and direct sequencing of viral material from clinical samples, i.e., often with low viral genetic loads and a high concentration of contaminant host nucleic acid background. To circumvent this difficulty, different techniques of enrichment can be achieved, either directly through laborious and time-consuming culture isolation or, even better, the use of specific primers targeting the whole genome of the virus (Houldcroft et al., 2017). Moreover, specific amplification-based approaches are proven successful since they can generate sufficient quantities of the genetic material needed for next-generation sequencing (Quick et al., 2017).

Recently, mobile next-generation sequencers have provided new opportunities in infectious diseases diagnostics and surveillance, such as the rapid sequencing of viral genomes during outbreaks. The pocket size nanopore technology sequencer MinION (Oxford Nanopore Technologies, ONT) is portable and field-deployable and enables real-time outbreak surveillance of threatening pathogens, such as Ebola, Lassa, and Zika viruses (Faria et al., 2016; Hoenen et al., 2016; Kafetzopoulou et al., 2019). Similarly, the recent Illumina iSeq100^TM^ system is the most compact, accessible, and affordable next-generation Illumina sequencer. This easy-to-use system is ideal for small whole-genome sequencing, e.g., viruses, and specific genomic targeted approaches (Colman et al., 2019).

In this study, we aimed at implementing an amplicon-based sequencing approach to obtain SARS-CoV-2 consensus genomes directly from clinical specimens, adaptable into the field conditions, with the two easily manageable next-generation sequencers, the nanopore MinION and the Illumina iSeq100^TM^ system. We also evaluated the performance of these two sequencers, in terms of ease of use, hands-on time, simplification of the sequencing process, and capacity for real-time genome sequencing in settings lacking laboratory resources for outbreak response.

## Materials and Methods

### Clinical Samples

The two samples used in this study were sputum (CIBU-200107 and CIBU-200132) collected from French suspected COVID-19 patients and addressed to the Laboratory for Urgent Response to Biological Threats for diagnostic testing. Sputum samples were handled in an enhanced biosafety level 2 laboratory, with full personal protective equipment (including FFP3 respirators) and procedures adapted to airborne pathogens, by trained staff, as recommended by WHO [Laboratory biosafety guidance related to coronavirus disease (COVID-19), interim guidance, 19 March 2020].

### Virus Culture

Vero E6 cells (mycoplasma-free) seeded in 24-well plates (4 × 10^5^ cells/well) were cultured in DMEM (Dulbecco’s modified Eagle medium; Thermo Fisher Scientific, United States) containing 1% PS (penicillin 10,000 U/ml; streptomycin 10,000 μg/ml) and supplemented with 5% FBS (fetal bovine serum). Samples were diluted 1:10 in DMEM 1% PS without FBS and supplemented with 1 μg/ml TPCK-trypsin (Sigma-Aldrich, United States), added to the cell monolayers and incubated for 1 h at 37°C in the presence of 5% CO_2_. The inoculum was then removed and replaced with fresh DMEM 1% PS containing 1 μg/ml TPCK-trypsin. After incubation for 3 days at 37°C in the presence of 5% CO_2_, cells were observed for the presence of a cytopathic effect (CPE) under the microscope, and culture supernatants were harvested, aliquoted, and stored at −80°C.

### Extraction of Genomic RNA From Specimens

Total RNA from clinical samples was extracted using the NucleoMag kit on the KingFisher automate (Macherey Nagel, Germany) and from isolates using the NucleoSpin DX Virus (Macherey Nagel, Germany), following the manufacturer’s instructions.

### Genome Detection by Real-Time RT-PCR

Sputum specimens and respective isolates RNA extracts were tested with the SARS-CoV-2 real-time *RdRP* gene duplex reverse transcription (RT)-PCR developed by the French National Reference Center for Respiratory Viruses and the real-time *E* gene RT-PCR from the Charité protocol (see WHO Coronavirus disease COVID-19 technical guidance: Laboratory testing for 2019-nCoV in humans, available from https://www.who.int/docs/default-source/coronaviruse/whoinhouseassays.pdf). Samples were considered SARS-CoV-2 positive if at least two out of three SARS-CoV-2 gene targets were detected by the RT-PCR assays.

### Amplicon-Based Protocol

**FIGURE 1:**
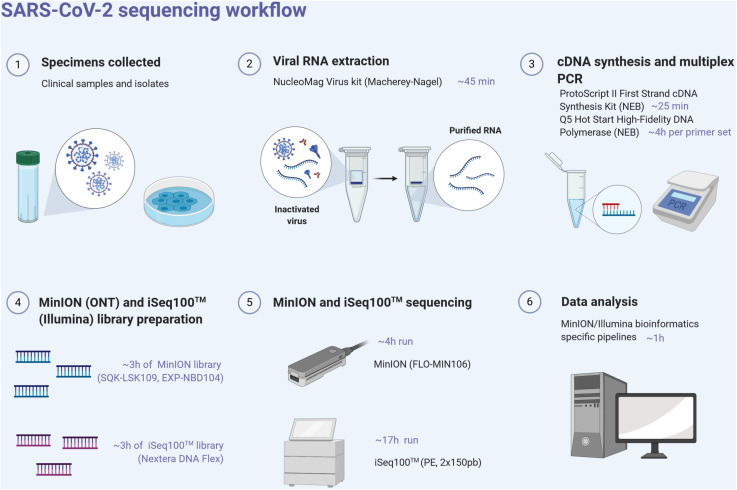
SARS-CoV-2 next-generation sequencing workflow, from the sample to sequence analysis. Created with biorender.com.

For each sample, the ProtoScript II First Strand cDNA synthesis kit (New England Biolabs, NEB, United States) was used to obtain single-strand cDNA from RNA extracts using random hexamers. RT was performed at 48°C for 15 min and 80°C for 5 min. Pools of specific primer sets were used to generate 36 amplicons from the cDNA using the Q5 Hot Start High-Fidelity DNA Polymerase (NEB, United States) (Supplementary Table). These multiplex primer sets (divided into two separate pools) were designed using the Primal scheme software^1^ on the SARS-CoV-2 reference sequence (Genbank accession number NC_045512) to sequentially amplify 1,000 bp using the following PCR conditions: 98°C for 30 s, 40 cycles of 98°C for 15 s, 65°C for 5 min, and ended by 65°C for 5 min. Clean-up of PCR products was performed with AMPure XP magnetic beads (Beckman Coulter, United States). The quantity of amplicons was measured with the Qubit 2.0 fluorometer using the dsDNA HS Assay Kit (Thermo Fisher Scientific, United States), and the quality was assessed by electrophoresis in E-Gel^®^ EX 1% agarose (Invitrogen^®^) and visualized with E-Gel PowerSnap Electrophoresis System (Invitrogen^®^). The amplicons obtained from the two clinical samples and their corresponding isolates were normalized to equal concentrations, multiplexed (X2), and sequenced using the MinION and Illumina iSeq100^TM^ System (Figure 1).

### MinION Sequencing

Library preparation for the MinION sequencing was performed using the Ligation Sequencing kit SQK-LSK109 and Natives Barcoding kits EXP-NBD104/EXP-NBD114 (ONT) according to the manufacturer’s instructions and modifications as per Quick et al. (2017). Briefly, PCR amplicons pools were end-repaired and dA-tailed using an UltraII End Prep Reaction Module (NEB, United States) followed by ligation of native barcodes using the NEBNext UltraII Ligation module (NEB, United States). All the pooled barcoded libraries were purified using AMpure XP beads (Beckman Coulter) followed by adapter ligation with the NEBNext UltraII Ligation module. Library clean-up was performed using AMpure XP beads and short fragment buffer (SFB) and then eluted in 15 μl of ONT’s elution buffer. The library was loaded onto an R9.4 flow cell (FLO-MIN106) and sequenced on a MinION Mk1B device within 4 h (Figure 1). ONT MinKNOW software (version 19.12.2) was used to collect raw data and perform basecalling (Guppy v3.4). The RAMPART tool (Read Assignment, Mapping, and Phylogenetic Analysis in Real Time) developed by the ARTIC network has been used to visualize genome coverage in real time and reference matching for each barcode.

### MinION Bioinformatics Workflow

From the fastq files, only reads with a minimum *Q* score of 7 were selected for the analysis. Data were demultiplexed using the guppy_barcoder with the option require_barcodes_both_ends to ensure barcodes are present at each end of the fragment. The reads were filtered on the expected length of amplicons. Reads between 900 and 1,200 base pairs were kept; thus, potential chimeric reads were removed. Selected reads were mapped against SARS-CoV-2 reference (NC_045512) using Minimap2 (v2.9) (Li, 2018). SAMtools (v1.9) were used to sort the aligned BAM files, to obtain coverage data and a consensus sequence. Alignment statistics were also calculated with SAMtools. Data were manually inspected using Tablet (v1.19) (Milne et al., 2013).

### Illumina iSeq100^TM^ Sequencing

The appropriate volume of each pool of amplicons was adjusted to have a total quantity of 100 ng of amplicon input (50 ng of each) per sample. The sequencing-ready libraries were prepared using the Nextera DNA Flex Prep kit (Illumina, United States). The libraries were qualified on an Agilent Technologies 2100 Bioanalyzer using a high-sensitivity DNA chip following the manufacturer’s instructions and quantified with the Qubit 2.0 fluorometer using the dsDNA HS Assay Kit (Thermo Fisher Scientific, United States). The resulting libraries were sequenced using the iSeq100^TM^ system (Illumina, United States) and in two multiplexed runs were performed generating 2 × 151 bp read length data during a 17 h run time (Figure 1).

### Illumina Bioinformatics Workflow

To remove low quality reads, trim off low-quality and contaminant residues, and filter out duplicated reads, fqCleaner v.0.5.0 was used, with Phred quality score of 28. Filtered reads were mapped against SARS-CoV-2 reference (NC_045512) using Burrows-Wheeler Aligner MEM algorithm (BWA-MEM) (v0.7.7). SAMtools (v1.9) was used to sort BAM files and to generate alignment statistics, coverage data, and a consensus sequence. Data were manually inspected using Tablet (v1.19) (Milne et al., 2013).

## Results

### Specimens’ Diagnostics

The two sputum samples CIBU-200107 and CIBU-200132, were tested with the specific SARS-CoV-2 real-time RT-PCR protocol. They were both found positive with the three SARS-CoV-2 gene targets. The threshold cycle (Ct) values for CIBU-200107 and CIBU-200132 were respectively 24-24 and 12-12 for the *RdRP* gene duplex and 25 and 13 for the *E* gene. The two clinical specimens were also inoculated on Vero E6 cells, and after incubation for 3 days, a clear cytopathic effect was observed. Culture supernatants of the first passage were harvested and tested for the presence of the virus with the specific SARS-CoV-2 real-time RT-PCR protocol. The Ct values of the two corresponding isolates CIBU-200107C1 and CIBU-200132C1 were respectively 21-21 and 10-10 for the *RdRP* gene duplex and 21 and 10 for the *E* gene, confirming the isolation of SARS-CoV-2.

### Amplicon-Based Protocol

As soon as the first SARS-CoV-2 reference genome was released in Genbank (NC_045512), we designed two sets of primers to generate a tiling path along the genome using the Primal Scheme tool. The full-length genome was amplified directly from the RNA extracts from the clinical samples and the corresponding isolates, to obtain PCR products overlapping by 120 bp and covering the entire 29,903 bp viral genomic sequence. Thirty-six amplicons, split into two pools, were successfully generated for each specimen with a size expected range between 900 and 1,098 bp (with an average length of 972 bp) and controlled by gel electrophoresis.

### MinION Sequencing

**FIGURE 2:**
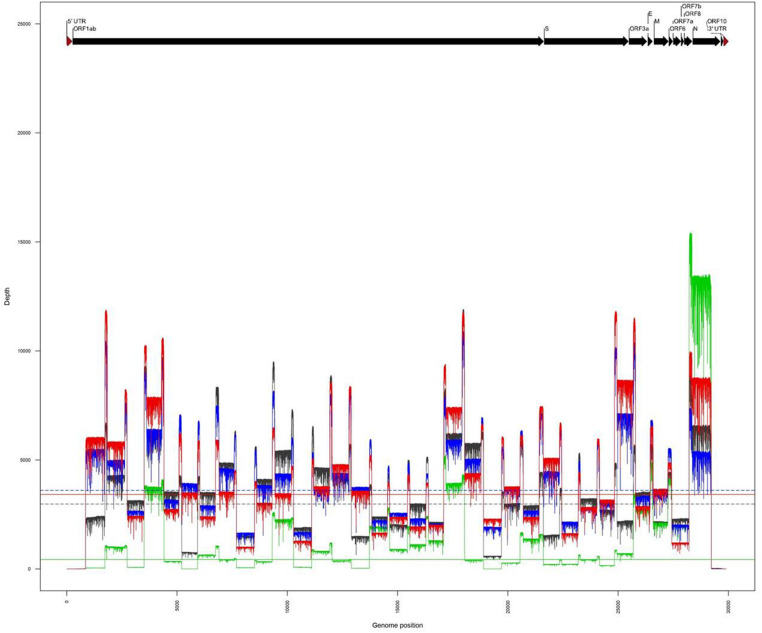
Comparison of genome coverage profiles obtained for the clinical samples (CIBU-200107 in green, CIBU-200132 in red) and their respective isolates (CIBU-200107C1 in black, CIBU-200132C1 in blue) using the MinION device. For each specimen, the corresponding median read depth is represented by horizontal lines; solid lines for clinical samples, and dashed lines for isolates, respectively.

The amplicons obtained from the two clinical samples CIBU-200107 and CIBU-200132 were multiplexed and sequenced using the MinION platform with R9.4.1 flow cell during 4 h. The MinION run produced 45,625 and 122,113 raw reads with a good quality score, respectively, for the two clinical samples, with an average of 1,074 bp read length. Thus, the mapping against Wuhan SARS-CoV-2 reference (NC_045512), by using Minimap2, retrieved 43,952 (96.3%) and 122,108 (99.9%) reads, respectively, for the two clinical samples (Table 1). The run yielded a median read depth of 436X and 3,418X for the two clinical samples, respectively (Table 1 and Figure 2), with the average base error rate ranging between 7.9 and 8.3%. A near full-length consensus sequence of 26,852 and 28,391 bp was generated for CIBU-200107 and CIBU-200132, respectively. In the same way, a 4 h MinION run, applied to the two corresponding isolates CIBU-200107C1 and CIBU-200132C1, produced 107,532 and 120,958 high-quality raw reads, respectively, with an average of 1,022 bp read length (Table 1). The run yielded a median read depth of 2,979X and 3,607X for the two isolates, respectively (Table 1 and Figure 2). The mapping against Wuhan SARS-CoV-2 reference (NC_045512) using Minimap2 used 99.9% of raw reads in both cases and allowed to generate the genome consensus sequence of 28,445 and 28,958 bp, for the two isolates, respectively. Regardless of using a read depth cutoff of 10X or 30X, we obtained around 89 and 94% of SARS-CoV-2 genome coverage, for the clinical samples, respectively. The genome coverage variation between both clinical samples reflected the initial viral load difference, which was even more marked at 100X (Table 1), as is the difference between the median read depths. However, due to the high sequencing error rate around 8%, we observed that a reliable consensus sequence should be analyzed at a minimum depth coverage at 30X, or even 100X for some nucleotides. Conversely, the genome coverage for the isolates was quite similar, with at 30X 94.9% and 95.3%, respectively (Table 1). For the clinical sample with the lower viral load CIBU-200107, the genome coverage was significantly improved by the isolation step, less so for the second clinical sample with a very low Ct value (Figure 2). Concerning the bioinformatics analysis, the RAMPART tool (Read Assignment, Mapping and Phylogenetic Analysis in Real Time) developed by the ARTIC network was helpful and time-saving to visualize genome coverage in real time and allowed to stop the run when sufficient data were generated. Globally, using our amplicon-based approach, combined with the MinION platform, we were able to obtain the near full-length genome of the studied viral specimens in around 8 h, from samples to sequences data.

**TABLE 1:** Overview of MinION and iSeq100^TM^ system sequences data obtained for both clinical specimens and corresponding isolates analyzed in this study.

	Nanopore MinION sequences		iSeq100^TM^ system sequences	

	Clinical sample	Isolate	Clinical sample	Isolate
**CIBU-200107**				
Total raw reads	45,625	107,532	3,990,761	3,770,873
Total mapped reads (% raw reads)	43,952 (96.3%)	107,529 (99.9%)	2,752,222 (69.0%)	3,764,987 (99.8%)
Average reads length	1,074	1,021	140	144
Base error rate	8.3%	7.9%	0.2%	0.1%
Median read depth	436X	2,979X	4,833X	20,054X
Genome coverage at reads depth 10X	89.8%	95.1%	94.7%	97.9%
Genome coverage at reads depth 30X	89.7%	94.9%	92.8%	97.8%
Genome coverage at reads depth 100X	76.0%	94.9%	89.5%	97.7%
**CIBU-200132**				
Total raw reads	122,113	120,958	4,046,340	3,790,945
Total mapped reads (% raw reads)	122,108 (99.9%)	120,956 (99.9%)	4,038,699 (99.8%)	3,786,372 (99.9%)
Average reads length	1,074	1,024	141	144
Base error rate	8.0%	7.9%	0.2%	0.1%
Median read depth	3,418X	3,607X	20,229X	19,163X
Genome coverage at reads depth 10X	94.9%	96.8%	97.8%	99.5%
Genome coverage at reads depth 30X	94.9%	95.3%	97.3%	98.7%
Genome coverage at reads depth 100X	94.9%	94.9%	96.7%	98.0%

### Illumina iSeq100^TM^ System Sequencing

**FIGURE 3:**
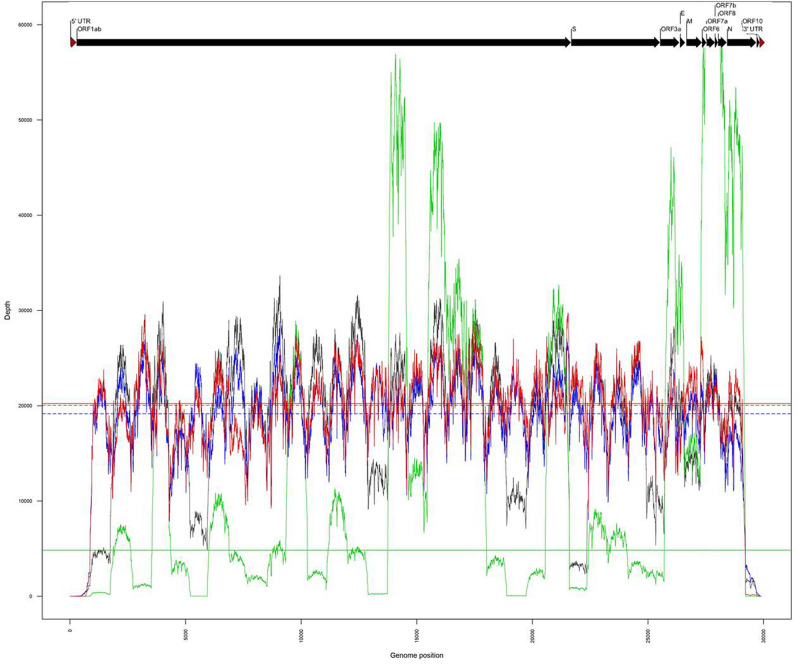
Comparison of genome coverage profiles obtained for the clinical samples (CIBU-200107 in green, CIBU-200132 in red) and their respective isolates (CIBU-200107C1 in black, CIBU-200132C1 in blue) using Illumina iSeq100^TM^ system. For each specimen, the corresponding median read depth is represented by horizontal lines; solid lines for clinical samples and dashed lines for isolates, respectively.

The same amplicons, obtained from the two clinical samples CIBU-200107 and CIBU-200132, were multiplexed and sequenced using the Illumina iSeq100^TM^ system during a run of 17 h. Illumina run generated 3,990,761 and 4,046,340 raw reads with a Phred quality score of 28, for the two clinical samples, respectively, with an average of 140 bp read length. The average base error rate was around 0.2%. The mapping against Wuhan SARS-CoV-2 reference (NC_045512), by using BWA-MEM, retrieved 2,752,222 (69%) and 4,038,699 reads (99.8%) for the two clinical specimens, respectively (Table 1). A near full-length consensus sequence of 28,243 and 29,253 bp was generated for the two clinical samples, respectively. Concerning the amplicons obtained from both isolates CIBU-200107C1 and CIBU-200132C1, they were also multiplexed and run independently in the same conditions. The run produced 3,770,873 and 3,790,945 of high-quality raw reads, with a very low base error rate of 0.1%, with an average of 144 bp read length (Table 1). By using BWA-MEM, the mapping against Wuhan SARS-CoV-2 reference (NC_045512) used almost all the produced raw reads (99.9%) in both cases and allowed to generate the genome consensus sequence of 29,283 and 29,727 bp, for the two isolates, respectively. With a read depth of 10X, 94.7 and 97.8% of genome coverage was obtained with a reliable consensus sequence, for the two clinical samples, respectively. Despite the viral load difference between the two clinical specimens, the quite high genome coverage is probably due to the low base error rate and the huge number of obtained reads. Finally, the median read depth (ranging from 19,163X to 20,229X) was quite similar for the high viral load clinical sample and both isolates, except CIBU-200107 with 4,833X median value (Figure 3). These observed variations in read depth are the direct consequence of the variable amplification efficiency of the primer pairs used in the multiplex PCR pool, impacted by the viral load of the samples as well as the secondary structures of the viral genome which affect primer binding. These results underlined that high-quality sequences could be obtained with greater samples multiplexing. Using an amplicon-based approach, combined with the iSeq100^TM^ system platform, we were able to obtain the near full genome of the studied SARS-CoV-2 in around 24 h.

### Mobile Next-Generation Sequencing Platforms Comparison for SARS-CoV-2 Genome Procurement

Viral consensus genomic sequences were rapidly and easily obtained for the two SARS-CoV-2 clinical specimens and their respective isolates, by using the two different sequencing platforms, MinION and iSeq100^TM^ system. We noted that the sequences obtained for the same specimen are identical, regardless of the sequencer used. Sequence analysis of the SARS-CoV-2 consensus genomes showed that they belong to the proposed phylogenetic clade A2^2^, which is consistent with the phylogenetic analysis of the other human French SARS-CoV-2 sequences (Gámbaro et al., 2020). Compared to the Wuhan reference sequence (NC_045512), the viral genomic sequences CIBU-200107 and CIBU-200132 (GISAID Accession numbers EPI_ISL_437689 and EPI_ISL_437690) display seven and six nucleotide mutations, respectively. Both share five nucleotide mutations (C241T, C3037T, C14408T, A23403G, and G25563T), among which three have led to amino acid mutations P4715L in ORF1ab, D614G in S, and Q57H in ORF3a. It is noteworthy that the mutation D614G in the Spike glycoprotein is specific of clade A2, encompassing most of the French SARS-CoV-2 sequences. The CIBU-200107 additionally contains two more mutations in ORF1ab, C1059T (T265I) and C10582T, whereas the CIBU-200132 includes an additional one, C2416T. Comparison of the genomes obtained for the clinical specimens and their respective isolates show that the sequences are strictly identical, suggesting that culture of the SARS-CoV-2 on Vero E6 cells did not induce any molecular change, at least during the first passage.

With many challenges still to be overcome in real-time genomics in rapid-response diagnostics, we evaluated the performances of these both mobile benchtop instruments. While the library preparation time is similar (approximately 3 h) between the two approaches and require the same benchtop laboratory equipment (Figure 1), the MinION is more efficient and versatile in terms of run time, with the procurement of robust results after 2–4 h, compared to an incompressible run time of 17 h for the iSeq100^TM^ system. It is important to take into account the thawing time of the Illumina cartridge reagents which takes at least 6 h in comparison to the immediately available MinION loading reagents which are stored at room temperature. MinION needs a powerful laptop computer and a stable broadband internet connection, whereas Illumina system is stand-alone at least in the first bioinformatics steps, as basecalling and demultiplexing. Concerning the sequencers themselves, the two systems are equivalent in terms of ease of use and maintenance and are therefore both suitable for experiments in resource-limited settings. The read length was fixed to 150 nt by the Illumina chemistry, and Nanopore reads were longer and entirely covered the length of the generated amplicons with an average of 1,074 nt, after trimming with Porechop (Table 1). While ONT improved sequencing accuracy, a still relatively high error rate of the MinION raw reads (around 8%) was noticed in comparison with Illumina ones (around 0.2%), requiring a higher read depth to obtain a confident consensus sequence. Overall, for each sample, we obtained a better coverage of the genome with the Illumina approach, and this as early as 10X of read depth. By comparing MinION and iSeq100^TM^ system produced consensus genomes, we observed that the necessary coverage threshold is higher for MinION than for iSeq100^TM^ system.

## Discussion

Novel mammalian coronaviruses are regularly identified and involved in epidemics and severe diseases, such as SARS-CoV, which emerged in southern China in 2002 (Drosten et al., 2003), and MERS-CoV, first described in Saudi Arabia in 2012 and still circulating (Song et al., 2019). At the source of viral outbreaks, readily adaptable methods with rapid turn-around times are necessary for pathogen identification, surveillance programs, and public health strategies. Whole genome sequencing with next-generation sequencers is now widely used in specialized laboratories, which have required equipment and long experience for this implementation. However, it is also essential that this approach is accessible at the pen-side of infected humans to investigate outbreaks in remote areas. In this regard, we therefore evaluated two space-saving and easily portable next-generation sequencers, MinION and iSeq100^TM^ system, through the current pandemic of COVID-19.

Although the initial investment gives an advantage to the MinION, the cost of reagents on the other hand is similar, and both approaches require the identical minimum of laboratory equipment to generate the libraries. By using the Primal scheme software on the first released SARS-CoV-2 reference sequence, we immediately developed an amplicon-based approach to sequence around 1,000 bp amplicons on our small portable sequencers. It has been reported that the ampliseq panel proposed by ARTIC network, based on 109 amplicons, may lead to coverage bias due to dimer formation between primers (Itokawa et al., 2020). Our choice to produce 36 longer amplicons, with lower number of primers, could be more efficient. Nevertheless, for all the specimens, the extremities of the genome could not be obtained. As often in the genome sequencing of single-strand RNA viruses, the 3’ and 5’ non-coding regions are quite difficult to directly acquire, which would rather require an approach such as rapid amplification of cDNA ends PCR (RACE-PCR).

We successfully proved that such targeted approach performs well directly with clinical samples. The counterpart of this workflow requires prior knowledge of the pathogen but, on the other hand, could be promptly adapted to all viruses of interest, including those with very large genomes. We found that viral genomic sequencing, even with low-throughput sequencers, could be performed with high confidence and be achieved directly from clinical specimens with significantly shortened run time. Although higher samples multiplexing is suitable, these relatively low throughput sequencers can also run economically with a small number of samples, which is a great advantage when the information is needed as quickly as possible. Both sequencers generated the correct consensus genomes, with a slightly lower coverage for MinION and a higher error rate than the iSeq100^TM^ system. However, we were able to obtain the genome of the studied specimens in around 8 h with the MinION, illustrating the quasi real-time sequencing capacity to effectively support health authorities. The main defect of the iSeq100^TM^ system finally lies in its run time. Nanopore is regularly considered to have the advantage over other technologies of high portability and fast turnaround time (Gardy and Loman, 2018). It should be noted that today Illumina offers a sequencer, which tends toward the same interesting features, while maintaining the high quality of the reads, which is at the origin of Illumina’s success. The MinION retains the advantage of greater versatility, with the possibility of analyzing data in true real time, particularly with tools such as RAMPART, and thus being able to stop the run after a few hours as soon as the amount of data is sufficient. Concerning the further generation of the alignment of amplicon reads against the reference and the variant calling, both devices usually offer tools in the cloud, such as Local Run Manager (DNA amplicon analysis module) for iSeq100^TM^ system, and EPI2ME for MinION. In outbreak situations, these two sequencers therefore make rapid sequencing accessible to a very large number of laboratories, requiring a minimum of molecular biology skills. Nevertheless, the improvement of the dedicated bioinformatics user-friendly resources should still be developed to take into account the low network and informatics resources in the field.

In conclusion, our investigation led to the development of an amplicon-based sequencing approach and the characterization of the viral genome as SARS-CoV-2 within a few hours, adaptable in field conditions, with two easily manageable next-generation sequencers, the nanopore MinION and the Illumina iSeq100^TM^ system. They provide faster and more cost-effective small-scale runs, which avoid outsourcing for laboratories in remote areas, and allow control of the whole sequencing process. The MinION and iSeq100^TM^ system bring the power of next-generation sequencing to virtually any laboratory, with amplicon-based whole-genome sequencing protocols rapidly transposable to all molecular investigations of epidemic prone pathogens.

## Data Availability Statement

The datasets generated for this study can be found in online repositories. The names of the repository/repositories and accession number(s) can be found in the article/Supplementary Material.

## Ethics Statement

Ethical review and approval was not required for the study on human participants in accordance with the local legislation and institutional requirements. Written informed consent for participation was not required for this study in accordance with the national legislation and the institutional requirements.

## Author Contributions

VH performed all the data analysis. AK and CB conducted the experiments. VH, AK, CB, JV, and VC conceived the study. VH, AK, CB, and VC analyzed the results and prepared the manuscript. SM, CB, and J-CM provided scientific guidance. All authors reviewed the manuscript.

## Conflict of Interest

The authors declare that the research was conducted in the absence of any commercial or financial relationships that could be construed as a potential conflict of interest.

## Footnotes

1 http://primal.zibraproject.org/

2 https://nextstrain.org/
