# Supplementary material for: Rapid Genomic Characterization of SARS-CoV-2 by Direct Amplicon-Based Sequencing Through Comparison of MinION and Illumina iSeq100TM System
Source: Front Microbiol. 2020 Sep 25;11:571328. doi: 10.3389/fmicb.2020.571328 (PMC7546329; doi:10.3389/fmicb.2020.571328)
Supplement: Supplementary file 1 [file Data_Sheet_1.PDF]

Primers List (Hourdel *et al.* 2020)

Primer Name	Primer Sequence	Pool
SARS-CoV-2_1_LEFT	ACATTCCCACCATACCTTCCCA	1
SARS-CoV-2_1_RIGHT	GTTCACGGCAGCAGTATACACC	1
SARS-CoV-2_2_LEFT	CCCTCTTGAGTGCATTAAAGACCT	2
SARS-CoV-2_2_RIGHT	TGTTCACCAATATTCCAGGCACC	2
SARS-CoV-2_3_LEFT	AACTGTGAAAGGTTTGGATTATAAAGCA	1
SARS-CoV-2_3_RIGHT	AGTAACCTTTGTTGGTGCACCG	1
SARS-CoV-2_4_LEFT	TGTTGCTCGAAATCAAAGACACAGA	2
SARS-CoV-2_4_RIGHT	GCCGACAACATGAAGACAGTGT	2
SARS-CoV-2_5_LEFT	CCATGCAAGTTGAATCTGATGATTACA	1
SARS-CoV-2_5_RIGHT	CATGTGCAAGCATTTCTCGCAA	1
SARS-CoV-2_6_LEFT	AGTGCTTAAAAAGTGTAAAAGTGCCT	2
SARS-CoV-2_6_RIGHT	TGCTGACATGTACCTACCCAGA	2
SARS-CoV-2_7_LEFT	AACCTCATAATTCACATGAAGGTAAAACA	1
SARS-CoV-2_7_RIGHT	GCTTGCGTTTGGATATGGTTGG	1
SARS-CoV-2_8_LEFT	ACAGAAATTGACCCTAAGTTGGACA	2
SARS-CoV-2_8_RIGHT	GCCTCTAGACAAAATTTACCGACACT	2
SARS-CoV-2_9_LEFT	AAACCGTGTTTGTACTAATTATATGCCTT	1
SARS-CoV-2_9_RIGHT	ACTGTAGTGACAAGTCTCTCGCA	1
SARS-CoV-2_10_LEFT	GCTTTTGCAAACTACACAATTGGAAT	2
SARS-CoV-2_10_RIGHT	GCAGCAACAAAAAGGAACACAAGT	2
SARS-CoV-2_11_LEFT	TGTTAATGTTGTAACAACAAAGATAGCACT	1
SARS-CoV-2_11_RIGHT	ACGATAGCTACAATACCACCAGCT	1
SARS-CoV-2_12_LEFT	TCTGTGGTGTAGATGCTGTAAATTTACT	2
SARS-CoV-2_12_RIGHT	CCCTGAGTTGAACATTACCAGCC	2
SARS-CoV-2_13_LEFT	TCTGCACCTCTGAAGACATGCT	1
SARS-CoV-2_13_RIGHT	GCTACAGTGGCAAGAGAAGGTAAC	1
SARS-CoV-2_14_LEFT	TGCTATGGGTATTATTGCTATGTCTGC	2
SARS-CoV-2_14_RIGHT	ACCCTGCATGGAAAGCAAAACA	2
SARS-CoV-2_15_LEFT	ATTGTGGGCTCAATGTGTCCAG	1
SARS-CoV-2_15_RIGHT	CCTACAAGGTGGTTCCAGTTCTG	1
SARS-CoV-2_16_LEFT	GGAGGTAGGTTTGTACTTGCACTG	2
SARS-CoV-2_16_RIGHT	AGCATAGACGAGGTCTGCCATT	2
SARS-CoV-2_17_LEFT	GCTGTTGCTAAACATGACTTCTTTAAGT	1
SARS-CoV-2_17_RIGHT	GTGCAGCTACTGAAAAGCACGT	1
SARS-CoV-2_18_LEFT	TTTTAAGGAATTACTTGTGTATGCTGCT	2
SARS-CoV-2_18_RIGHT	TGCATTAACATTGGCCGTGACA	2
SARS-CoV-2_19_LEFT	TGGCGGTTCACTATATGTTAAACCA	1
SARS-CoV-2_19_RIGHT	ACAACCTGGAGCATTGCAAACA	1
SARS-CoV-2_20_LEFT	TGCATACGTAGACCATTCTTATGTTGT	2
SARS-CoV-2_20_RIGHT	TCACATAGTGCATCAACAGCGG	2
SARS-CoV-2_21_LEFT	GGTAAGAGTCATTTTGCTATTGGCC	1
SARS-CoV-2_21_RIGHT	TGTAAAGTTGCCACATTCCTACGT	1
SARS-CoV-2_22_LEFT	GCTATTACCAGAGCAAAAGTAGGCA	2
SARS-CoV-2_22_RIGHT	CAAAGCACTCGTGGACAGCTAG	2
SARS-CoV-2_23_LEFT	ACAGGTAACCTACAAAGCAACCA	1
SARS-CoV-2_23_RIGHT	TGGTACTGGTTTAATGTTGCGCT	1
SARS-CoV-2_24_LEFT	ACACAAAAGTTGATGGTGTTGATGT	2
SARS-CoV-2_24_RIGHT	GATTAGGCATAGCAACACCCGG	2

SARS-CoV-2_25_LEFT	TCTGTAGTTTCTAAGGTTGTCAAAGTGA	1
SARS-CoV-2_25_RIGHT	ACACACTGACTAGAGACTAGTGGC	1
SARS-CoV-2_26_LEFT	AGGGGTACTGCTGTTATGTCTTTAAA	2
SARS-CoV-2_26_RIGHT	GGTCAAGTGCACAGTCTACAGC	2
SARS-CoV-2_27_LEFT	GTGCTGCAGCTTATTATGTGGGT	1
SARS-CoV-2_27_RIGHT	ACGGACAGCATCAGTAGTGTCA	1
SARS-CoV-2_28_LEFT	TGGTTTAACAGGCACAGGTGTT	2
SARS-CoV-2_28_RIGHT	TGAGCAAAGGTGGCAAAACAGT	2
SARS-CoV-2_29_LEFT	TGCAGATGCTGGCTTCATCAAA	1
SARS-CoV-2_29_RIGHT	ACAACATCACAGTTACCAGACACA	1
SARS-CoV-2_30_LEFT	AGGTGTCTTTGTTTCAAATGGCAC	2
SARS-CoV-2_30_RIGHT	CATTTCCAGCAAAGCCAAAGCC	2
SARS-CoV-2_31_LEFT	GCCTTGAAGCCCCTTTTCTCTA	1
SARS-CoV-2_31_RIGHT	ACTAGGTTCCATTGTTCAAGGAGC	1
SARS-CoV-2_32_LEFT	TCCTGATCTTCTGGTCTAAACGAACT	2
SARS-CoV-2_32_RIGHT	AGCGAGTGTTATCAGTGCCAAG	2
SARS-CoV-2_33_LEFT	TTATGAGGACTTTTAAAGTTTCCATTTGGA	1
SARS-CoV-2_33_RIGHT	ACTGCCAGTTGAATCTGAGGGT	1
SARS-CoV-2_34_LEFT	AGAGTATCATGACGTTCGTGTTGT	2
SARS-CoV-2_34_RIGHT	CGACATTCCGAAGAACGCTGAA	2
SARS-CoV-2_35_LEFT	GTGGTCCAGAACAAACCCAAGG	1
SARS-CoV-2_35_RIGHT	TTGGCAGAGACACTTGAGTTGC	1
SARS-CoV-2_36_LEFT	TCCTGCTGCAGATTTGGATGAT	2
SARS-CoV-2_36_RIGHT	CAAAAAGATGCCATAGCCCTTTGC	2
